# Supplementary material for: The interactome of intact mitochondria by cross-linking mass spectrometry provides evidence for coexisting respiratory supercomplexes
Source: Mol Cell Proteomics. 2017 Dec 8;17(2):216–32. doi: 10.1074/mcp.RA117.000470 (PMC5795388; doi:10.1074/mcp.RA117.000470)
Supplement: Supplemental Data [file supp_RA117.000470_133922_0_supp_23978_fzffwf.zip › spectra_annotation/mito_DR_spectra_annotation/443-1-4-1-7-1.pdf]

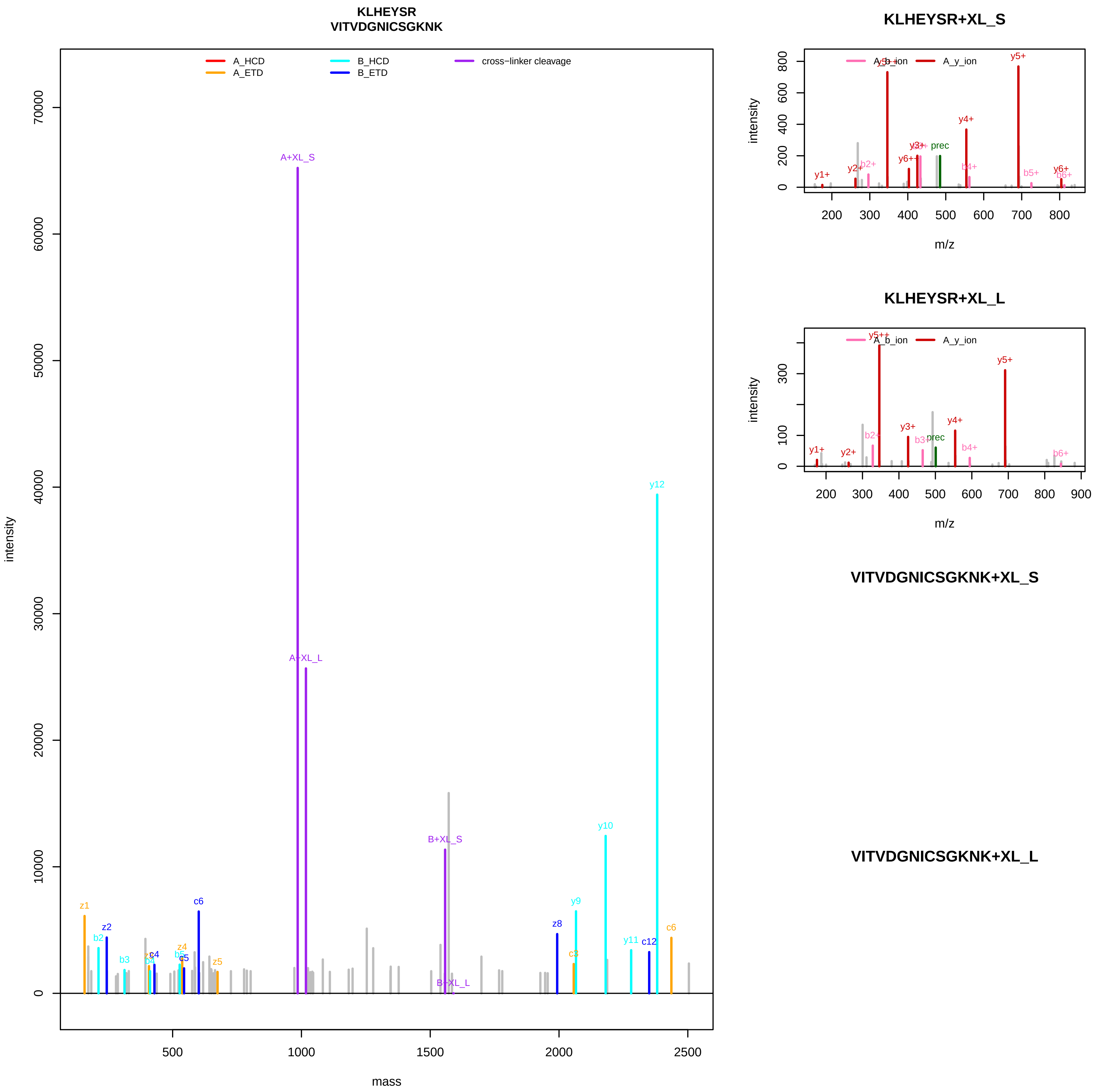
KLHEYSR
VITVDGNICSGKNK
A_HCD
A_ETD
B_HCD
B_ETD
cross−linker cleavage
A+XL_S
A+XL_L
B+XL_S
B+XL_L
y12
y10
y9
y11
c12
z8
c3
c6
z1
b2
z2
b3
c4
b4
z4
b5
c5
c6
z5
intensity
mass
KLHEYSR+XL_S
KLHEYSR+XL_L
A_b_ion
A_y_ion
prec
y5+
y5++
y4+
y3+
y6++
y2+
b2+
y1+
b4+
b5+
y6+
b6+
b3+
m/z
VITVDGNICSGKNK+XL_S
VITVDGNICSGKNK+XL_L
